# Present and future screening programs for diabetic retinopathy: a narrative review

**DOI:** 10.1186/s40942-024-00534-8

**Published:** 2024-02-03

**Authors:** Andreas Abou Taha, Sebastian Dinesen, Anna Stage Vergmann, Jakob Grauslund

**Affiliations:** 1 Department of Ophthalmology, Odense University Hospital, Sdr. Boulevard 29, 5000 Odense, Denmark; 2 Department of Clinical Research, University of Southern Denmark, Odense, Denmark; 3 Steno Diabetes Center Odense, Odense University Hospital, Odense, Denmark

**Keywords:** Artificial intelligence, Diabetic retinopathy, Screening program, Epidemiology

## Abstract

Diabetes is a prevalent global concern, with an estimated 12% of the global adult population affected by 2045. Diabetic retinopathy (DR), a sight-threatening complication, has spurred diverse screening approaches worldwide due to advances in DR knowledge, rapid technological developments in retinal imaging and variations in healthcare resources.

Many high income countries have fully implemented or are on the verge of completing a national Diabetic Eye Screening Programme (DESP). Although there have been some improvements in DR screening in Africa, Asia, and American countries further progress is needed. In low-income countries, only one out of 29, partially implemented a DESP, while 21 out of 50 lower-middle-income countries have started the DR policy cycle. Among upper-middle-income countries, a third of 59 nations have advanced in DR agenda-setting, with five having a comprehensive national DESP and 11 in the early stages of implementation.

Many nations use 2–4 fields fundus images, proven effective with 80–98% sensitivity and 86–100% specificity compared to the traditional seven-field evaluation for DR. A cell phone based screening with a hand held retinal camera presents a potential low-cost alternative as imaging device. While this method in low-resource settings may not entirely match the sensitivity and specificity of seven-field stereoscopic photography, positive outcomes are observed.

Individualized DR screening intervals are the standard in many high-resource nations. In countries that lacks a national DESP and resources, screening are more sporadic, i.e. screening intervals are not evidence-based and often less frequently, which can lead to late recognition of treatment required DR.

The rising global prevalence of DR poses an economic challenge to nationwide screening programs AI-algorithms have showed high sensitivity and specificity for detection of DR and could provide a promising solution for the future screening burden.

In summary, this narrative review enlightens on the epidemiology of DR and the necessity for effective DR screening programs. Worldwide evolution in existing approaches for DR screening has showed promising results but has also revealed limitations. Technological advancements, such as handheld imaging devices, tele ophthalmology and artificial intelligence enhance cost-effectiveness, but also the accessibility of DR screening in countries with low resources or where distance to or a shortage of ophthalmologists exists.

## Background

Diabetic retinopathy (DR) is a recognized sight-threatening complication to diabetes and is recommended for screening by the World Health Organization [1–3]. Various studies confirm the cost-effectiveness of DR screening despite variations in approaches and the availability of diverse imaging technologies across different countries [1, 4].

The imperative for DR screening is expected to escalate concomitantly with the rising prevalence of diabetes [5]. As a consequence, it is crucial to elucidate the current status of DR screening at a global scale. This narrative review is necessary to assess the prevailing levels of awareness, accessibility and implementation of DR screening programs worldwide. Understanding the existing landscape will provide insight into the adequacy of the current screening measures and highlight areas that may require enhancement to effectively address the growing prevalence of diabetes and its associated ocular complications.

This narrative review aims to provide a global perspective of DR epidemiology and screening while exploring new approaches alongside development of artificial intelligence (AI) technology.

## Methods

### Data sources

This narrative review aims to be as comprehensive as possible in identifying data. The sources used for identification of literature were MEDLINE, Embase and The Cochrane Database of Systematic reviews. We used the search terms “diabetic retinopathy”, “screening of diabetic retinopathy”, “prevalence of diabetic retinopathy”, “incidence of diabetic retinopathy”, “artificial intelligence”, “deep learning” and obtained information on ongoing DR screening programs, not published in scientific journals, from the official pages of the World Health Organization and the International Diabetes Federation.

### Inclusion criteria

Studies and reports focusing on epidemiology and screening programs for DR were considered for inclusion. A comprehensive approach involved the inclusion of both cross-sectional and longitudinal studies to investigate the prevalence of DR, but we considered only longitudinal studies for exploration of DR and PDR incidences. Inclusion criteria encompassed studies that (1) examined national or subnational DR screening programs regardless of economic status, (2) investigated epidemiology of DR and (3) were written in English.

## Global epidemiology of DR

### Prevalence

**Fig. 1:**
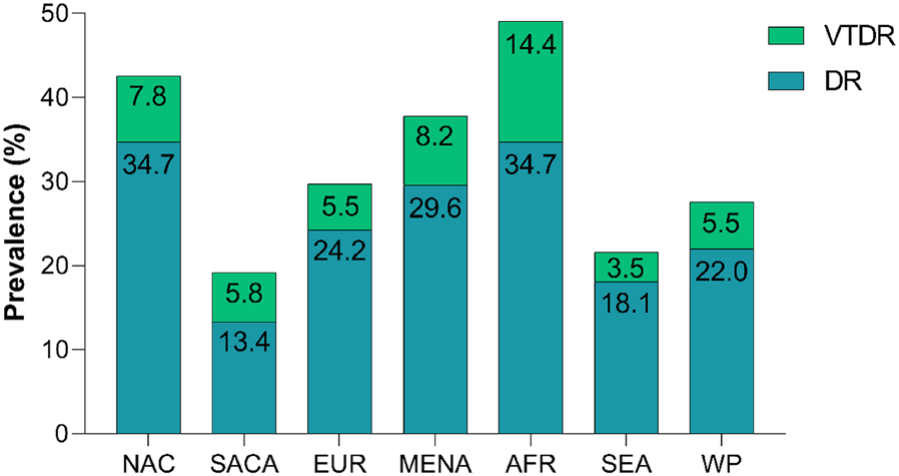
The figure illustrates the global prevalence of diabetic retinopathy, including vision-threatening cases, based on populations-based systematic reviews [6, 7]. The proportion of vision-threatening cases are highlighted above the overall prevalence of diabetic retinopathy. *DR* diabetic retinopathy, *VTDR* vision threatening diabetic retinopathy. *NAC* North America and Caribbean, *SACA* South and Central America, *EUR* Europe, *MENA* Middle East and Northern Africa, *AFR* Africa, SEA South East Asia, *WP* Western Pacific

It is estimated that diabetes affects 783 million people aged 20–79 years worldwide by 2045, which equals 12.2% of the global adult population [5]. As life expectancy continues to rise and prevalence of diabetes increases, the prevalence of DR is expected to rise alongside [6]. Figure 1 displays the global prevalence of DR and vision threatening DR in both type 1 and 2 diabetes according to data from two comprehensive systematic reviews [6, 7].

### Incidence of DR in type 1 diabetes

Most studies investigating the incidence rates of DR in patients with type 1 diabetes are of older date [8–10]. These include a study by Klein et al. from 1989 that reported a 4 year incidence of DR in type 1 diabetes of 59.0% [8], a European study from 1986 that reported a 5 year incidence of 47.0% [9] and a Swedish study from 2003 that found a 10 year incidence of 39% [10].

### Incidence of DR in type 2 diabetes

A Danish cohort study of patients with type 2 diabetes who attended the Danish screening program for DR showed a 5 year incidence of 3.8% for DR [11] compared to 4% in a study from United Kingdom (UK) [12]. A group from India reported a 4 year incidence of 9.2% [13], while studies of older date from Hong Kong [14], Australia [15] and USA [16] reported substantially higher 5 year incidences of 15.2, 22.2 and 38.6%, respectively.

### Incidence of PDR

**Fig. 2:**
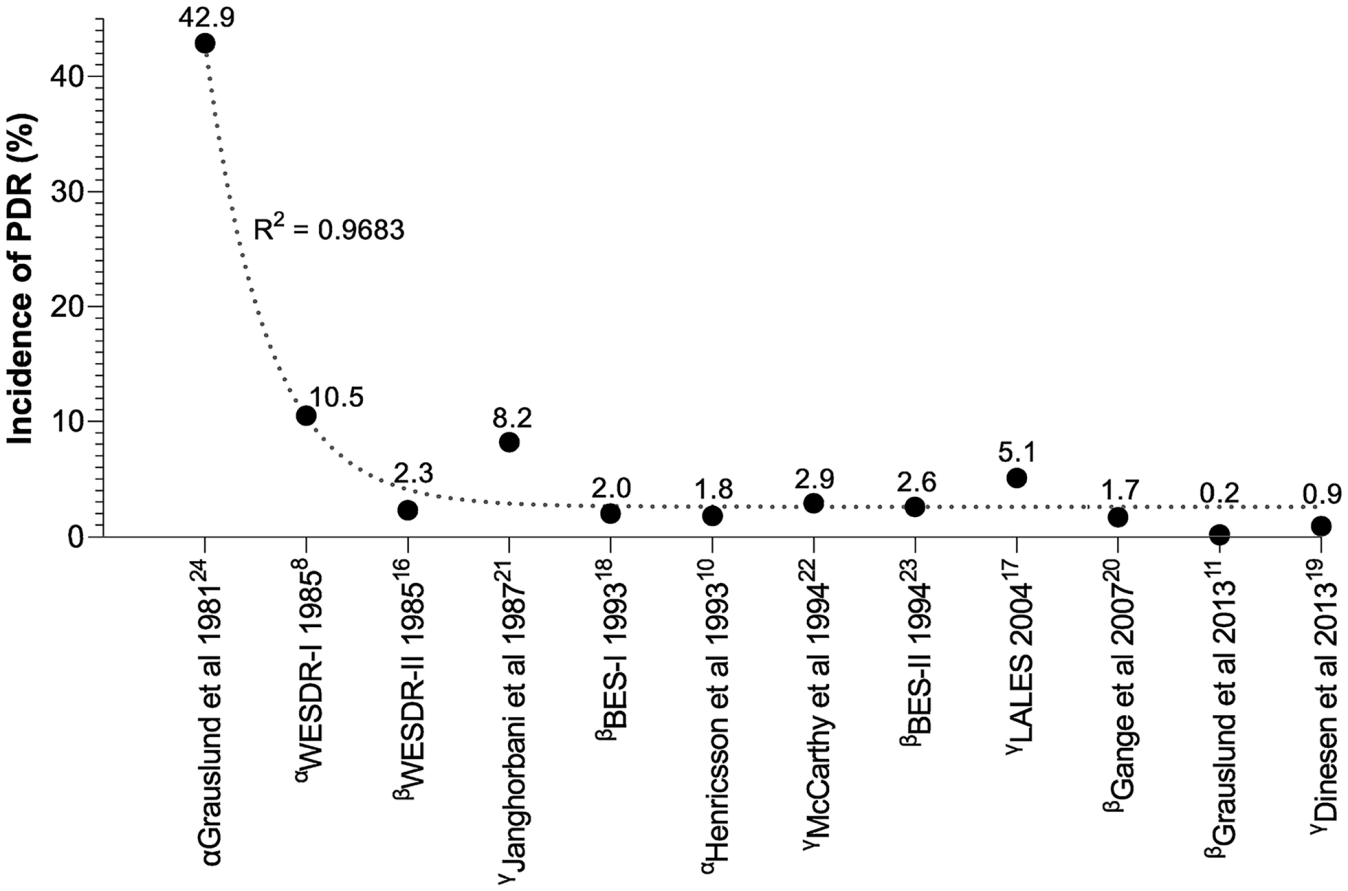
Trends in the incidence of proliferative diabetic retinopathy in population-based studies of type 1^α^ or 2 diabetes^β^ and some including both types of diabetes^γ^. The year marks the baseline date of each follow-up period and the uppercase number is the reference number. *PDR* proliferative diabetic retinopathy

We identified 11 population-based studies that investigated the incidence of PDR over various follow-up periods of four [8, 16–18], five [11, 19–22], nine [23], ten [10] and 25 years [24]. Among these studies, three focused on patients with type 1 diabetes [8, 10, 24], five on patients with type 2 diabetes [11, 16, 18, 20, 23], while four studies encompassed populations that comprised patients with both type 1 and 2 diabetes [17, 19, 21, 22]. The incidences of PDR are displayed in Fig. 2 arranged chronologically based on the year marking the baseline date. Starting with the earliest study and progressing to the latest, the line of tendency shows that the incidence of PDR has significantly declined over the 32 year period.

## Current DR screening recommendations

Countries like Iceland, UK, Ireland, and Denmark have national diabetic eye screening programmes (DESP) [1, 4]. Many nations are making considerable progress in developing regional screening and treatment services including Norway, Sweden, the Netherlands, Czech Republic, Italy, Poland, Serbia, Hungary, Turkey and others that can be studied in Table 1 [4].

**Table 1:** A global overview of nations with a national or partially implemented diabetic retinopathy screening program (DESP)

National level of income	Nation	National or partially implemented DESP
Low-income nations	None found	
Lower-middle-income nations		
	Bangladesh [26]	Partial
	India [66, 67]	Partial
	Kenya [68]	Partial
	Nepal [69]	Partial
	Pakistan [70]	Partial
	Peru [28]	Partial
	Sri Lanka [71]	Partial
	Tanzania [72]	Partial
	Vietnam [73]	Partial
	West Bank and Gaza [74]	Partial
	Zambia [75]	Partial
Higher-middle-income nations		
	Argentina [33]	Partial
	Bosnia and Herzegovina [76]	Partial
	Botswana [77]	National
	Brazil [34]	National
	China [78]	Partial
	Costa Rica [29]	Partial
	Fiji [79]	Partial
	Lebanon [80]	Partial
	Malaysia [81]	Partial
	Mexico [30, 31]	Partial
	South Africa [82]	Partial
	Thailand [83]	Partial
High-income nations		
	Australia [84]	Partial
	Bahrain [85]	Partial
	Canada [86]	Partial
	Chile [87]	Partial
	Croatia [35]	Partial
	Denmark [1]	National
	Estonia [88]	Partial
	Finland [89]	National
	France [90]	Partial
	Germany [91]	Partial
	Iceland [92]	National
	Ireland [93]	National
	Italy [94]	Partial
	Malta [95]	National
	New Zealand [96]	National
	Norway [97]	Partial
	Northern Ireland [98]	National
	Portugal [99]	Partial
	Scotland [100]	National
	Singapore [101]	Partial
	Slovenia [102]	National
	South Korea [103]	Partial
	Spain [104]	Partial
	Sweden [105, 106]	Partial
	The Netherlands [107]	Partial
	UK [42, 108]	National
	USA [37–40]	Partial

A consultative group of the International Agency for the Prevention of Blindness (IAPB) categorized 10 South-East Asia countries (SEAC) into low (Myanmar and Timor-Leste), middle (Bhutan, Indonesia, Maldives, Myanmar, Nepal and Sri Lanka) and high resource (Thailand and India) level and made recommendations of DR management [25]. Even though only four of these countries (India, Nepal, Sri Lanka and Thailand) have developed national DR guidelines, the middle resource countries have made improvements in DR screening due to the increasing prevalence of diabetes and its complication [25, 26].

However, DR screening in SEACs, like many low-, lower-middle- and upper-middle-income countries that lacks a national DESP, is sporadic and the methods are either screening camps, telemedicine vans, opportunistic screening, or physician-led screenings utilizing direct ophthalmoscopy, where only a small number of patients undergoes mydriatic imaging [26, 27]. A review authored by Vujosevic et al. [4], observed advancements in DR screening in African and Asian countries, which included Botswana, China, Singapore, Indonesia, and Bangladesh.

In examining the management of DR in various Middle- and South American countries, a disparity in resource allocation and healthcare provision become evident [28–32].

Peru has established a comprehensive DR referral network in La Libertad, but still faces challenges in in impeding widespread screening and treatment, due to limited resources, thus categorizing it as a low-resource nation [28].

The middle-resource countries Argentina [33], Brazil [34], Costa Rica [29], and Mexico [30, 31] demonstrates different stages of progress in DR screening and management. However, they share common struggles in achieving uniformity and comprehensive access in DR care [32]. Argentina's particular challenge lies in its highly distorted economy with persistently high inflation and a massive fiscal deficit, which had led to economic constraints and had impacted the national healthcare strategies for diabetes and DR [33]. On the other hand, Mexico [30, 31] and Costa Rica [29] both underscore the need for improved healthcare strategies and effective disease management.

A study from Costa Rica [29] showed that 23.5% of individuals with diabetes had retinopathy and/or maculopathy, with 6.2% having Vision-Threatening DR. The study urges the need for improvement, especially among the older population, in DR screening methods and management e.g. the authors suggests that conventional screening methods like direct ophthalmoscopy have low sensitivity and may not be as effective.

Another study found that the coverage of DR screening among diabetic patients in Brazil [34] has increased from 12.1% in 2014 to 21.2% in 2019. Nevertheless, it was concluded that further progress is required in these regions, due to the fact that screenings of DR often are private insurance-based health care, decentralized health care screening, or has not been expanded from localized regions to encompass the entire nation, and therefore with significant regional differences [34].

The studies illustrate the broader challenge in the Latin American context, where the growth in DR screening is yet to meet the needs dictated by the prevalence and complexity of the condition.

SEAC, Middle- and South American countries need more widespread access to trained staff in order to have a fully functional DESP [4, 35] e.g. Thailand face challenges due to the long distance to specialized medical practices, while China is challenged with only 20 ophthalmologists per one million people in contrast to 49 in the UK and 59 in the USA. [36].

The USA also faces challenges due to the fact that DR screening differs across different states and are insurance-based. Many health insurance plans, including the national Medicare, typically cover annual diabetic eye exams, but not all insurances cover more intensive follow-up or economic loss due to less work that particular day, which can lead to the screening being influenced by personal economics instead of evidence based recommendations [37–40].

Curran et al. [41] found that of 29 identified low-income countries, only four had data available on DR policy planning, and just one had a partially rolled out DESP. Among the 50 lower-middle-income countries, 21 had begun a DR policy cycle, with a single nation having a national DESP and 18 with DESPs in the early implementation phase. For Upper-Middle-Income Countries, 22 out of 59 countries had advanced in DR agenda-setting. Only five of these Upper-Middle-Income Countries had a comprehensive national DESP in place with 11 more in the partially implemented stages of DESP.

## Practical approaches

### Visual acuity

Several DESPs incorporate the assessment of visual acuity as a part of the screening routine [1, 42], but is not sufficiently sensitive to stand alone [43]. This limitation arises from the fact that a significant number of patients may remain asymptomatic until vision threatening DR manifests, often precipitated by vitreous bleeding or clinically significant diabetic macula edema.

### Classification scales

Several classification systems of DR exist e.g. English National Screening Programme -, Wisconsin Diabetic Retinopathy—and the Scottish Diabetic retinopathy grading system. The gold standard in classification of DR in clinical trials has traditionally been the Early Treatment of Diabetic Retinopathy Study (ETDRS) classification scale [2, 44, 45]. This is an evidence based approach to screening and have demonstrated its effectiveness in predicting the risk of progression to proliferative DR and vision loss. However, the ETDRS scale use in a clinical settings is limited due to its complexity and many levels of DR classification. A simplified version of the ETDRS system, the International Clinical Diabetic Retinopathy (ICDR) scale [46], is recommended by various international clinical guidelines, which includes the guidelines established by the International Council of Ophthalmology (ICO) for everyday clinical practice [2]. Therefore, several nations use the ICDR severity scale in DR screening worldwide. The ICDR severity scale categorise DR into following levels accordingly to the severity of DR.

Level 0 is the absence of DR. Level 1 is mild non-proliferative DR (NPDR) characterized exclusively by microaneurysms and/or dot haemorrhages. Level 2 representing moderate NPDR, which is defined as more severe than level 1 but less than level 3. Level 3 indicates severe NPDR, where there’s observed more than 20 intraretinal haemorrhages in each of the four quadrant, or definite venous beading in at least 2 quadrants or prominent intraretinal microvascular abnormalities in at least 1 quadrant, but no proliferative DR. Level 4: signifies proliferative DR [46].

### Standard fundus images

The gold standard for evaluating DR in clinical trials has traditionally been ETDRS seven-standard fields, which are a compilation of seven stereoscopic 30-degree fundus images [44, 45]. A review from 2020 [4] found that a limited number of fundus images (typically two to four) exhibit a sensitivity ranging from 80 to 98% and a specificity between 86 and 100% when compared to the results obtained from ETDRS seven-fields in detecting DR. Conversely, a single central field was found to have lower sensitivity (ranging from 54 to 78%) and specificity (between 88 and 89%) when compared to the results of the ETDRS seven-standard fields.

The use of limited single-field fundus photos [4] is found to be effective, especially considering the difficulties, expenses, and time constraints associated with performing the ETDRS seven-standard fields, making it impractical for routine screening [1]. As a result, most Western nations rely on the simplicity and efficiency of limited single-field fundus photos, covering around 30% of the retinal surface [42].

The Danish and UK guidelines recommend a minimum two-field mydriatic fundus photos for DR screening. The retinal images should encompass a minimum horizontal field of view of 45° in the UK and 70–80° in Denmark. The vertical coverage should be at least 40° in the UK and 45° in Denmark [1, 42].

The recommendation from the IAPB in SEAC is that low resource SEACs uses a minimum of four-field non-mydriatic fundus photos with a 30° camera, whereas middle- and high resource SEACs uses minimum two-field non-mydriatic fundus photos with wide-field (50°or more) camera [25].

### Alternative image modalities

A recent alternative technique has emerged, known as the cell phone-based approach. This method involves utilizing a handheld condensing lens in combination with a smartphone camera to capture retinal images [47–49].

A review [4] conducted in Western Australia, where handheld retinal cameras were introduced for community-based clinical assessments of DR in low-resource settings, demonstrated positive result and thereby showed a potential for such systems to expand eye care services to underserved areas and remote locations.

No handheld devices have yet matched the sensitivity and specificity of seven-field stereoscopic photography in detecting sight-threatening DR. Rajalakshmi et al. [49] conducted a comparative study in which they evaluated the performance of a smartphone-based retinal camera against seven-field digital retinal photography. It was found that these methods produced identical results in 92.7% of patients, with a substantial kappa statistic of 0.90. Jacoba et al. [50] discovered that depending on the referral threshold, up to 37.0% of individual eyes with PDR might remain undetected when utilizing handheld photos.

This aligns with the result of several studies [51, 52] which found high agreement in DR classification and image quality between handheld fundus cameras with standard tabletop fundus cameras for DR. However, disagreements in microaneurysms, small hemorrhages, and intraretinal microvascular abnormalities, contributed to the higher discordance within non-proliferative DR due to the decreased resolution in the retinal microvasculature. Moreover, the studies found that for referable DR and vision-threatening DR the agreement was 85% with only a substantial kappa statistic of 0.7. Consequently, they recommended lowering the referral thresholds to an eye-centre when utilizing handheld devices.

On the other hand, DR detection in smartphone-based fundus photography using AI [52–54] showed high sensitivity and specificity in detecting DR and sight-threatening DR, suggesting that AI-based smartphone retinal imaging could be a valuable tool for mass retinal screening in diabetes.

### Screening intervals

The ICO recommends that the interval between DR screenings varies from 1 month to 2 years depending on the patients DR severity according to the ICDR Severity Scale. In recent years national guidelines for DR screening in several Western countries have shifted from fixed or sporadic screening intervals to a more individualized approach. These updated guidelines seek to optimize healthcare resources by adjusting screening intervals according to individualized risks of DR progression. Factors like glycaemic control and blood pressure are taken into account, leading to shorter or longer screening intervals, even when patients fall within the same severity group on the ICDR scale [1, 42, 55].

In SEACs patients with mild non-proliferative diabetic retinopathy (NPDR), the recommended screening interval according to IAPB is one year. This is the same for moderate NPDR [27]. Another study [32] recommend screening interval of 2 years in case of no- and mild NPDR in low- and middle income countries, and 1 year interval for moderate NPDR. In the case of proliferative DR, once the condition is stabilized, the recommended interval is six months [26, 27, 32].

## Automated retinal image analysis

The availability of resources for nationwide screening programs remains limited in many countries. Nevertheless, advancements in technology have emerged as game-changers in screening strategies, enhancing cost-effectiveness. Technologies like scanning confocal ophthalmology with UWF, handheld mobile devices, tele ophthalmology for remote grading, and AI for automated detection and classification of DR have transformed screening approaches. These innovations are not only improving the efficiency of screening, but also contributing to better cost-effectiveness outcomes [4].

Four reviews [36, 56–58] refers to numerous studies where deep learning (DL) algorithms have consistently demonstrated remarkable high sensitivity, specificity, and area under the receiver operating characteristic curve for detection of DR, Diabetic macula edema and other eye conditions like glaucoma as well as age-related macular degeneration (AMD).

Cheung et al. [56] suggests that the use of DL technology could reduce costs and improve access while enhancing patient outcomes through early detection and treatment of DR. Ballemo et al. [36] highlights that studies on DL have been conducted across various countries, showing promising results. The obstacles related to current DR screening and the motivations for implementing AI differ across nations.

The motivation for adopting AI in countries with well-established healthcare systems like the UK and Singapore is to sustain a high-quality healthcare service for patients while optimizing available resources [36].

AI could be an instrument capable of enhancing screening availability to people in lower-middle-income countries, but also to countries with long distances to specialized medical practices, and to countries with low numbers of ophthalmologists per million people [36, 57]. Studies have looked at AI for photo analysis of fundus photo taken by handheld smartphone devices at patients with dilated pupils [54, 59, 60]. Using a four-fundus image [59], a two-image [54], and a single-image [60] approach showed good results with automatic AI screening, even though Penha et al. [60] emphasize that it has been established that a single image protocol loses diagnostic accuracy in comparison to a two-image protocol when it comes to expert human reading. However, with automatic reading using AI systems, the performance of a single image protocol was considered satisfactory for screening.

Cheung et al. [56] and Bellemo et al. [36] propose a model where retinal images will be firstly analysed by the DL systems. If the system does not find the need for further intervention, the patients will be rescanned accordingly to the screening program. However, if the system finds the need for intervention, two possible options are proposed: a semi-automated model where the images will be read by doctors or trained graders before referring the patients to an eye-centre or a fully-automated model where patients will be referred to an eye-centre without further investigations. Both models will lower the number of images doctors or trained graders should analyse, but could also increase the amount of patients that undergoes screening per day.

## Discussion

The progress made in imaging, treatment and understanding of DR has disrupted the existing DR screening guidelines, giving rise to diverse practical approaches to DR screening in various countries.

A recent review from 2021 [32] underscores the global variations in DR screening practices. It stated that developed countries focus on an effort to enhance the effectiveness and accuracy of DESPs, like optimized screening intervals and adoption of new imaging technologies. In contrast, for the vast majority of world countries, especially those with limited resources, the primary challenge lies in establishing basic DR screening infrastructures. The focus in these countries is therefore making basic DR screening accessible and efficient.

In numerous low-, lower- and middle-income nations, the primary challenge hindering the effectiveness of screening programs is the absence of DESP, insufficient resources, and limited access to skilled healthcare professionals [27]. Furthermore, the individual financial situations can influence the screening process, screening frequency, and access to treatment.

Screening is a vital step in disease management, and can potentially reduce the disease burden according to The World Health Organization [61]. Particularly in low-, lower- and middle-income nations this is more complex, because screening may improve disease detection but doesn’t always lead to adequate treatment. Thus, for screening programs to be truly impactful, they must be integrated with effective treatment strategies to ensure a meaningful decrease in the burden of diseases and to prevent serious consequences [61].

For countries that have fully implemented or are nearing the completion of a national DESP, opportunities for further enhancements still exist. Denmark has implemented a nationwide DR screening program in 2013 and the guidelines for this program have been grounded in comprehensive evidence-based practices since 2018 [1]. The screening in Denmark [2, 42] is conducted by private practicing ophthalmologists and hospitals ensuring comprehensive coverage. A recent Danish study [62] revealed an impressive overall agreement of 93% between inter-grader reliability within the Danish screening program for DR. These results highlight the reliability and consistency of grading outcomes within the Danish screening program for DR.

Another potential area of improvement, as demonstrated by the Danish screening program, is the aspect where the screening results are documented in the Danish Registry of Diabetic Retinopathy (DiaBase), which is a national clinical quality database [63]. This feature enables the program to make informed decisions and adapt its screening strategies based on the data collected. For instance, a cohort study conducted in 2022 [64] examined participants in the Danish screening program from 2013 to 2018. The study determined the characteristics of patients who experienced delays in attending screenings and evaluated the impact of these delays on the progression of DR.

Shortcomings in these evidence-based guidelines does on the other hand exist. These guidelines focus solely on the vascular aspect of the disease and disregard evaluation of neural retina and diabetic retinal neurodegeneration. Scientific evidence has suggested that early neural degeneration may precede or coexist with vascular lesions and impact visual function [47]. The existing guidelines fails to account for the regression or resolution of retinal neovascularization as well as the influence of retinal areas with ischaemia or hypo perfusion.

There have been expression of concerns that the screening relies on limited standard field photographs (seven-field or less) [2, 46]. The issue with fundus photos covering a “limited part” of the retinal surface is the risk of exclusion of peripheral retinal lesions, which could have prognostic implications that can enhance outcome prediction. New imaging technologies like UWF can capture approximately 82% of the retinal surface. Studies using UWF imaging also showed that around 50% of neovascularization cases are predominantly peripheral [47, 48]. However, Kernt et al. [55] and Silva et al. [65] showed that UWF imaging rarely resulted in better detection of peripheral lesions in eyes with PDR that would not have been detected otherwise with ETDRS seven-standard fields. With the growing prevalence of diabetes [5] the demand for cost-effective DR screenings is substantial. Inadequate resources allocated to advanced imaging technologies, trained professionals or specialized facilities, might hinder accessibility and timely diagnosis, leading to increased long-term healthcare expenses due to untreated DR complications [1, 3, 4, 56, 57]. The implementation of AI in screening for DR can potentially enhance the cost-effectiveness, however this raises ethical concerns such as data privacy, algorithmic bias, and patient consent, which need to be addressed. Addressing issue like transparent AI algorithms, safeguarding patient data, and maintaining human oversight in decision-making are necessary steps to overcome these concerns [56]. The black box phenomenon is a returning concern because clinicians and patients always seek a reason for conclusions. This lack of understanding of how the algorithm comes to its conclusions, makes it impossible for physicians to detect potential biases. The issue of responsibility in the event of adverse patient outcomes due to AI-based technology is also a critical concern of many physicians [36, 56].

DL algorithms demand substantial data to achieve acceptable performance levels. However, countries with lower income are often underrepresented in these datasets, which can lead to less accurate DL algorithms for these populations. Moreover “real-world" experiments are vital for validating DL algorithms and minimize bias [36, 56]. Lower income countries may lack the resources required for developing, implementing, and retraining, which can decrease the benefits of AI based DR screening [57].

Many healthcare systems are not able to share data due to the protection of sensitive personal information, and the availability for diverse datasets from various settings and populations are limited [56]. In the realm of AI applied to DR screening, it is imperative for researchers to diligently pursue collaborative initiatives centred on data sharing. This concerted effort is essential to strengthen generalizability of AI models, thereby fortifying the safety and efficacy of forthcoming advancements in this research domain.

## Conclusion

This review sheds light on the need for effective DR screening programs, especially with the projected rise in diabetes cases worldwide. While some countries have well-established national screening programs, many struggle with decentralized or private-based healthcare systems and the need of broader access to trained professionals, which all impact screening effectiveness.

Screening approaches, including practical classification scales, individualize screening intervals and new imaging modalities shows promising results, but also limitations. The emergence of AI technology raises hope for countries to either continues high standards DR screening without enormous economic cost, or enhancing screening accessibility to their inhabitants. However ethical concerns and the need for robust validation of AI persist.

## Data Availability

Not applicable. Data sharing is not applicable to this article as no datasets were generated or analysed during the current study.

## Abbreviations

DR: Diabetic retinopathy
PDR: Proliferative diabetic retinopathy
AI: Artificial intelligence
UK: United Kingdom
DESP: Diabetes eye screening programs
IAPB: International Agency for the Prevention of Blindness
SEAC: South-East Asia countries
ETDRS: Early Treatment of Diabetic Retinopathy Study
ICDR: International Clinical Diabetic Retinopathy Scale
ICO: International Council of Ophthalmology
NPDR: Non-proliferative Diabetic Retinopathy

## References

[1] Grauslund J, Andersen N, Andresen J, Flesner P, Haamann P, Heegaard S (2018). Evidence-based Danish guidelines for screening of diabetic retinopathy. Acta Ophthalmol.

[2] Wong TY, Sun J, Kawasaki R, Ruamviboonsuk P, Gupta N, Lansingh VC (2018). Guidelines on diabetic eye care: the international council of ophthalmology recommendations for screening, follow-up, referral, and treatment based on resource settings. Ophthalmology.

[3] Lanzetta P, Sarao V, Scanlon PH, Barratt J, Porta M, Bandello F (2020). Fundamental principles of an effective diabetic retinopathy screening program. Acta Diabetol.

[4] Vujosevic S, Aldington SJ, Silva P, Hernández C, Scanlon P, Peto T (2020). Screening for diabetic retinopathy: new perspectives and challenges. Lancet Diabetes Endocrinol.

[5] Sun H, Saeedi P, Karuranga S, Pinkepank M, Ogurtsova K, Duncan BB (2022). IDF diabetes atlas: global, regional and country-level diabetes prevalence estimates for 2021 and projections for 2045. Diabetes Res Clin Pract.

[6] Teo ZL, Tham YC, Yu M, Chee ML, Rim TH, Cheung N (2021). Global prevalence of diabetic retinopathy and projection of burden through 2045: systematic review and meta-analysis. Ophthalmology.

[7] Hashemi H, Rezvan F, Pakzad R, Ansaripour A, Heydarian S, Yekta A (2022). Global and regional prevalence of diabetic retinopathy; a comprehensive systematic review and meta-analysis. Semin Ophthalmol.

[8] Klein R, Klein BE, Moss SE, Davis MD, DeMets DL (1989). The wisconsin epidemiologic study of diabetic retinopathy. IX. Four-year incidence and progression of diabetic retinopathy when age at diagnosis is less than 30 years. Arch Ophthalmol.

[9] Burger W, Hövener G, Düsterhus R, Hartmann R, Weber B (1986). Prevalence and development of retinopathy in children and adolescents with type 1 (insulin-dependent) diabetes mellitus. A longitudinal study. Diabetologia.

[10] Henricsson M, Nyström L, Blohmé G, Ostman J, Kullberg C, Svensson M (2003). The incidence of retinopathy 10 years after diagnosis in young adult people with diabetes: results from the nationwide population-based diabetes incidence study in Sweden (DISS). Diabetes Care.

[11] Grauslund J, Pedersen FN, Andersen N, Andresen J, Bek T, Dinesen S (2023). Presence and development of diabetic retinopathy in 153 238 patients with type 2 diabetes in the Danish registry of diabetic retinopathy. Acta Ophthalmol.

[12] Jones CD, Greenwood RH, Misra A, Bachmann MO (2012). Incidence and progression of diabetic retinopathy during 17 years of a population-based screening program in England. Diabetes Care.

[13] Raman R, Ganesan S, Pal SS, Gella L, Kulothungan V, Sharma T (2017). Incidence and progression of diabetic retinopathy in Urban India: Sankara Nethralaya-diabetic retinopathy epidemiology and molecular genetics study (SN-DREAMS II), report 1. Ophthalmic Epidemiol.

[14] Song H, Liu L, Sum R, Fung M, Yap MK (2011). Incidence of diabetic retinopathy in a Hong Kong Chinese population. Clin Exp Optom.

[15] Cikamatana L, Mitchell P, Rochtchina E, Foran S, Wang JJ (2007). Five-year incidence and progression of diabetic retinopathy in a defined older population: the blue mountains eye study. Eye.

[16] Klein R, Klein BE, Moss SE, Davis MD, DeMets DL (1989). The wisconsin epidemiologic study of diabetic retinopathy. X. Four-year incidence and progression of diabetic retinopathy when age at diagnosis is 30 years or more. Arch Ophthalmol.

[17] Varma R, Choudhury F, Klein R, Chung J, Torres M, Azen SP (2010). Four-year incidence and progression of diabetic retinopathy and macular edema: the Los Angeles Latino eye study. Am J Ophthalmol.

[18] Leske MC, Wu SY, Hennis A, Nemesure B, Hyman L, Schachat A (2003). Incidence of diabetic retinopathy in the Barbados eye studies. Ophthalmology.

[19] Dinesen S, Stokholm L, Subhi Y, Peto T, Savarimuthu TR, Andersen N (2023). Five-year incidence of proliferative diabetic retinopathy and associated risk factors in a nationwide cohort of 201 945 Danish patients with diabetes. Ophthalmol Sci.

[20] Gange WS, Lopez J, Xu BY, Lung K, Seabury SA, Toy BC (2021). Incidence of proliferative diabetic retinopathy and other neovascular sequelae at 5 years following diagnosis of type 2 diabetes. Diabetes Care.

[21] Janghorbani M, Jones RB, Allison SP (2000). Incidence of and risk factors for proliferative retinopathy and its association with blindness among diabetes clinic attenders. Ophthalmic Epidemiol.

[22] McCarty DJ, Fu CL, Harper CA, Taylor HR, McCarty CA (2003). Five-year incidence of diabetic retinopathy in the melbourne visual impairment project. Clin Exp Ophthalmol.

[23] Leske MC, Wu SY, Hennis A, Nemesure B, Schachat AP, Hyman L (2006). Nine-year incidence of diabetic retinopathy in the Barbados eye studies. Arch Ophthalmol.

[24] Grauslund J, Green A, Sjølie AK (2009). Prevalence and 25 year incidence of proliferative retinopathy among Danish type 1 diabetic patients. Diabetologia.

[25] Takkar B, Das T, Thamarangsi T, Rani PK, Thapa R, Nayar PD (2022). Development of diabetic retinopathy screening guidelines in South-East Asia region using the context, challenges, and future technology. Semin Ophthalmol.

[26] Muqit MMK, Kourgialis N, Jackson-deGraffenried M, Talukder Z, Khetran ER, Rahman A (2019). Trends in diabetic retinopathy, visual acuity, and treatment outcomes for patients living with diabetes in a fundus photograph-based diabetic retinopathy screening program in Bangladesh. JAMA Netw Open.

[27] Ramasamy K, Raman R, Tandon M (2013). Current state of care for diabetic retinopathy in India. Curr Diab Rep.

[28] Salamanca O, Geary A, Suárez N, Benavent S, Gonzalez M (2018). Implementation of a diabetic retinopathy referral network. Peru Bull World Health Organ.

[29] Acevedo Castellón RI, Carranza Vargas E, Cortés Chavarría RE, Rodríguez Vargas GA (2019). Rapid assessment of avoidable blindness and diabetic retinopathy in individuals aged 50 years or older in Costa Rica. PLoS ONE.

[30] Polack S, Yorston D, López-Ramos A, Lepe-Orta S, Baia RM, Alves L (2012). Rapid assessment of avoidable blindness and diabetic retinopathy in Chiapas, Mexico. Ophthalmology.

[31] Barquera S, Campos-Nonato I, Aguilar-Salinas C, Lopez-Ridaura R, Arredondo A, Rivera-Dommarco J (2013). Diabetes in Mexico: cost and management of diabetes and its complications and challenges for health policy. Global Health.

[32] Das T, Takkar B, Sivaprasad S, Thanksphon T, Taylor H, Wiedemann P (2021). Recently updated global diabetic retinopathy screening guidelines: commonalities, differences, and future possibilities. Eye.

[33] Caporale JE, Elgart JF, Gagliardino JJ (2013). Diabetes in argentina: cost and management of diabetes and its complications and challenges for health policy. Global Health.

[34] Fernandes AG, Ferraz AN, Brant R, Malerbi FK (2022). Diabetic retinopathy screening and treatment through the Brazilian National Health Insurance. Sci Rep.

[35] Loncarek K, Petricek I, Brajac I, Filipović T, Vatavuk Z, Stalekar H (2005). Quality of screening for diabetic retinopathy in the Rijeka region of Croatia. Coll Antropol.

[36] Bellemo V, Lim G, Rim TH, Tan GSW, Cheung CY, Sadda S (2019). Artificial intelligence screening for diabetic retinopathy: the real-world emerging application. Curr Diab Rep.

[37] Silva PS, Cavallerano JD, Tolls D, Omar A, Thakore K, Patel B (2014). Potential efficiency benefits of nonmydriatic ultrawide field retinal imaging in an ocular telehealth diabetic retinopathy program. Diabetes Care.

[38] Fransen SR, Leonard-Martin TC, Feuer WJ, Hildebrand PL (2002). Clinical evaluation of patients with diabetic retinopathy: accuracy of the Inoveon diabetic retinopathy-3DT system. Ophthalmology.

[39] Cavallerano JD, Silva PS, Tolson AM, Francis T, Tolls D, Patel B (2012). Imager evaluation of diabetic retinopathy at the time of imaging in a telemedicine program. Diabetes Care.

[40] Kirkizlar E, Serban N, Sisson JA, Swann JL, Barnes CS, Williams MD (2013). Evaluation of telemedicine for screening of diabetic retinopathy in the Veterans Health Administration. Ophthalmology.

[41] Curran K, Piyasena P, Congdon N, Duke L, Malanda B, Peto T (2023). Inclusion of diabetic retinopathy screening strategies in national-level diabetes care planning in low- and middle-income countries: a scoping review. Health Res Policy Syst.

[42] Scanlon PH (2017). The english national screening programme for diabetic retinopathy 2003–2016. Acta Diabetol.

[43] Scanlon PH, Foy C, Chen FK (2008). Visual acuity measurement and ocular co-morbidity in diabetic retinopathy screening. Br J Ophthalmol.

[44] Early photocoagulation for diabetic retinopathy (1991). ETDRS report number 9. Early treatment diabetic retinopathy study research group. Ophthalmology.

[45] Fundus photographic risk factors for progression of diabetic retinopathy (1991). ETDRS report number 12. Early treatment diabetic retinopathy study research group. Ophthalmology.

[46] Wilkinson CP, Ferris FL, Klein RE, Lee PP, Agardh CD, Davis M (2003). Proposed international clinical diabetic retinopathy and diabetic macular edema disease severity scales. Ophthalmology.

[47] Yang Z, Tan TE, Shao Y, Wong TY, Li X (2022). Classification of diabetic retinopathy: past, present and future. Front Endocrinol.

[48] Gangwani RA, Lian JX, McGhee SM, Wong D, Li KK (2016). Diabetic retinopathy screening: global and local perspective. Hong Kong Med J.

[49] Rajalakshmi R, Arulmalar S, Usha M, Prathiba V, Kareemuddin KS, Anjana RM (2015). Validation of smartphone based retinal photography for diabetic retinopathy screening. PLoS ONE.

[50] Jacoba CMP, Salongcay RP, Aquino LAC, Salva CMG, Saunar AV, Alog GP (2023). Comparisons of handheld retinal imaging devices with ultrawide field images for determining diabetic retinopathy severity. Acta Ophthalmol.

[51] de Oliveira JAE, Nakayama LF, Zago Ribeiro L, de Oliveira TVF, Choi S, Neto EM (2023). Clinical validation of a smartphone-based retinal camera for diabetic retinopathy screening. Acta Diabetol.

[52] Rajalakshmi R, Prathiba V, Arulmalar S, Usha M (2021). Review of retinal cameras for global coverage of diabetic retinopathy screening. Eye.

[53] Karakaya M, Hacisoftaoglu RE (2020). Comparison of smartphone-based retinal imaging systems for diabetic retinopathy detection using deep learning. BMC Bioinformatics.

[54] Malerbi FK, Andrade RE, Morales PH, Stuchi JA, Lencione D, de Paulo JV (2022). Diabetic retinopathy screening using artificial intelligence and handheld smartphone-based retinal camera. J Diabetes Sci Technol.

[55] Kernt M, Hadi I, Pinter F, Seidensticker F, Hirneiss C, Haritoglou C (2012). Assessment of diabetic retinopathy using nonmydriatic ultra-widefield scanning laser ophthalmoscopy (Optomap) compared with ETDRS 7-field stereo photography. Diabetes Care.

[56] Cheung CY, Tang F, Ting DSW, Tan GSW, Wong TY (2019). Artificial intelligence in diabetic eye disease screening. Asia Pac J Ophthalmol.

[57] Cleland CR, Rwiza J, Evans JR, Gordon I, MacLeod D, Burton MJ (2023). Artificial intelligence for diabetic retinopathy in low-income and middle-income countries: a scoping review. BMJ Open Diabetes Res Care.

[58] Nielsen KB, Lautrup ML, Andersen JKH, Savarimuthu TR, Grauslund J (2019). Deep learning-based algorithms in screening of diabetic retinopathy: a systematic review of diagnostic performance. Ophthalmol Retina.

[59] Rajalakshmi R, Subashini R, Anjana RM, Mohan V (2018). Automated diabetic retinopathy detection in smartphone-based fundus photography using artificial intelligence. Eye.

[60] Penha FM, Priotto BM, Hennig F, Przysiezny B, Wiethorn BA, Orsi J (2023). Single retinal image for diabetic retinopathy screening: performance of a handheld device with embedded artificial intelligence. Int J Retina Vitreous.

[61] Dans LF, Silvestre MA, Dans AL (2011). Trade-off between benefit and harm is crucial in health screening recommendations. Part I: general principles. J Clin Epidemiol.

[62] Thykjaer AS, Andresen J, Andersen N, Bek T, Heegaard S, Hajari J (2023). Inter-grader reliability in the Danish screening programme for diabetic retinopathy. Acta Ophthalmol.

[63] Andersen N, Hjortdal J, Schielke KC, Bek T, Grauslund J, Laugesen CS (2016). The Danish registry of diabetic retinopathy. Clin Epidemiol.

[64] Thykjær AS, Andersen N, Bek T, Heegaard S, Hajari J, Laugesen CS (2022). Attendance in a national screening program for diabetic retinopathy: a population-based study of 205,970 patients. Acta Diabetol.

[65] Silva PS, Cavallerano JD, Sun JK, Soliman AZ, Aiello LM, Aiello LP (2013). Peripheral lesions identified by mydriatic ultrawide field imaging: distribution and potential impact on diabetic retinopathy severity. Ophthalmology.

[66] Murthy GVS, Gilbert C, Shukla R, Bala V, Anirudh GG, Mukpalkar S (2020). Overview and project highlights of an initiative to integrate diabetic retinopathy screening and management in the public health system in India. Indian J Ophthalmol.

[67] Gaiha SM, Shukla R, Gilbert CE, Anchala R, Gudlavalleti MV (2016). Is India's policy framework geared for effective action on avoidable blindness from diabetes?. Indian J Endocrinol Metab.

[68] Mwangi N, Ng'ang'a M, Gakuo E, Gichuhi S, Macleod D, Moorman C (2018). Effectiveness of peer support to increase uptake of retinal examination for diabetic retinopathy: study protocol for the DURE pragmatic cluster randomized clinical trial in Kirinyaga, Kenya. BMC Public Health.

[69] Bhatta S, Pant N, Pant SR (2023). Prevalence of diabetes and diabetic retinopathy in far-western province of Nepal. J Nepal Health Res Counc.

[70] Bechange S, Roca A, Schmidt E, Gillani M, Ahmed L, Iqbal R (2021). Diabetic retinopathy service delivery and integration into the health system in Pakistan-findings from a multicentre qualitative study. PLoS ONE.

[71] Piyasena M, Murthy GVS, Yip JLY, Gilbert C, Peto T, Premarathna M (2019). A qualitative study on barriers and enablers to uptake of diabetic retinopathy screening by people with diabetes in the Western Province of Sri Lanka. Trop Med Health.

[72] Hall CE, Hall AB, Mallya J, Courtright P, Kok G (2022). Establishing a screening programme for diabetic retinopathy in Kilimanjaro Region, Tanzania using intervention mapping. Eye.

[73] Curran K, Lohfeld L, Congdon N, Peto T, Hoang TT, Nguyen HT (2022). Ophthalmologists' and patients' perspectives on treatments for diabetic retinopathy and maculopathy in Vietnam: a descriptive qualitative study. BMJ Open.

[74] Yahya T, Nazzal Z, Abdul-Hadi AR, Belkebir S, Hamarshih M, Fuqaha A (2020). Diabetic retinopathy screening barriers among Palestinian primary health care patients: a qualitative study. J Diabetes Metab Disord.

[75] Lewis AD, Hogg RE, Chandran M, Musonda L, North L, Chakravarthy U (2018). Prevalence of diabetic retinopathy and visual impairment in patients with diabetes mellitus in Zambia through the implementation of a mobile diabetic retinopathy screening project in the Copperbelt province: a cross-sectional study. Eye.

[76] Pidro A, Ahmedbegovic-Pjano M, Grisevic S, Sofic-Drino V, Gabric K, Biscevic A (2019). Epidemiology of diabetic retinopathy at eye clinic Svjetlost Sarajevo: two years retrospective single center study. Mater Sociomed.

[77] Blake A, Munby HN, Katlego PM, Sebuyuyu PM, Nkomozana O, Bangure R, Kerr-Muir M, Ngondi J (2015). Characteristics of patients with diabetic retinopathy in Gaborone. Botswana Tanzania Health Res Bullet.

[78] You QS, Xu L, Wang YX, Liang QF, Cui TT, Yang XH (2013). Prevalence of diabetic retinopathy as cause for visual impairment: the Beijing public health care project. Clin Exp Ophthalmol.

[79] Ram S, Mohammadnezhad M, Ram K, Prasad K, Pal M, Dalmia P (2022). Increasing and sustaining diabetic retinopathy screening in Fiji by leveraging community health workers (CHWs) services: a qualitative study. Heliyon.

[80] Chelala E, Saleh N, Dirani A, Fadlallah A, Baz P, Slim E (2015). Screening of diabetic retinopathy and maculopathy in Lebanese population using retinography and SD-OCT: The role of telemedicine. J Med Liban.

[81] Ngah NF, Muhamad NA, Asnir ZZ, Abdul Aziz RA, Mhad Kassim Z, Sahar SA (2020). Descriptive assessment on diabetic retinopathy screening in an awareness programme in Malaysia. Int J Ophthalmol.

[82] Abdool Z, Naidoo K, Visser L (2022). Development of a diabetic retinopathy screening model for a district health system in Limpopo Province, South Africa. Afr Vis Eye Health.

[83] Ruamviboonsuk P, Tiwari R, Sayres R, Nganthavee V, Hemarat K, Kongprayoon A (2022). Real-time diabetic retinopathy screening by deep learning in a multisite national screening programme: a prospective interventional cohort study. Lancet Digit Health.

[84] Murray RB, Metcalf SM, Lewis PM, Mein JK, McAllister IL (2005). Sustaining remote-area programs: retinal camera use by Aboriginal health workers and nurses in a Kimberley partnership. Med J Aust.

[85] Al Alawi E, Ahmed AA (2012). Screening for diabetic retinopathy: the first telemedicine approach in a primary care setting in Bahrain. Middle East Afr J Ophthalmol.

[86] Maberley D, Walker H, Koushik A, Cruess A (2003). Screening for diabetic retinopathy in James Bay, Ontario: a cost-effectiveness analysis. CMAJ.

[87] Arenas-Cavalli JT, Abarca I, Rojas-Contreras M, Bernuy F, Donoso R (2022). Clinical validation of an artificial intelligence-based diabetic retinopathy screening tool for a national health system. Eye.

[88] Krieger B, Hallik R, Kala K, Ülper K, Polonski M (2022). Validation of mobile-based funduscope for diabetic retinopathy screening in Estonia. Eur J Ophthalmol.

[89] Laitinen A, Laatikainen L, Härkänen T, Koskinen S, Reunanen A, Aromaa A (2010). Prevalence of major eye diseases and causes of visual impairment in the adult Finnish population: a nationwide population-based survey. Acta Ophthalmol.

[90] Massin P, Chabouis A, Erginay A, Viens-Bitker C, Lecleire-Collet A, Meas T (2008). OPHDIAT: a telemedical network screening system for diabetic retinopathy in the Ile-de-France. Diabetes Metab.

[91] Kreft D, McGuinness MB, Doblhammer G, Finger RP (2018). Diabetic retinopathy screening in incident diabetes mellitus type 2 in Germany between 2004 and 2013—a prospective cohort study based on health claims data. PLoS ONE.

[92] Kristinsson JK, Hauksdóttir H, Stefánsson E, Jónasson F, Gíslason I (1997). Active prevention in diabetic eye disease. A 4-year follow-up. Acta Ophthalmol Scand.

[93] Diabetic Retina Screen: The National Diabetic Retinal Screening Programme. https://www.diabeticretinascreen.ie/home/default.aspx?id=2&amprcss=print.

[94] Scarpa G, Urban F, Vujosevic S, Tessarin M, Gallo G, Visentin A (2016). The nonmydriatic fundus camera in diabetic retinopathy screening: a cost-effective study with evaluation for future large-scale application. J Ophthalmol.

[95] Diabetes: A National Public Health Priority. A National Strategy for Diabetes. 2016.

[96] Zealand HN. Diabetic Retinal Screening, Grading, Monitoring and Referral Guidance. 2016.

[97] Carlsen S, Skrivarhaug T, Thue G, Cooper JG, Gøransson L, Løvaas K (2017). Glycemic control and complications in patients with type 1 diabetes—a registry-based longitudinal study of adolescents and young adults. Pediatr Diabetes.

[98] Jamison C, Peto T, Quinn N, D'Aloisio R, Cushley LN, Johnston PC (2021). Screening attendance, prevalence and severity of diabetic retinopathy (DR) in a cohort of patients with diabetes mellitus secondary to chronic pancreatitis (DMsCP) in Northern Ireland. BMJ Open Diabetes Res Care.

[99] Pereira AMP, da Silva Laureano RM, de Lima Neto FB (2021). Five regions, five retinopathy screening programmes: a systematic review of how Portugal addresses the challenge. BMC Health Serv Res.

[100] Looker HC, Nyangoma SO, Cromie DT, Olson JA, Leese GP, Black MW (2014). Rates of referable eye disease in the Scottish national diabetic retinopathy screening programme. Br J Ophthalmol.

[101] Tan TE, Wong TY (2022). Diabetic retinopathy: looking forward to 2030. Front Endocrinol.

[102] Gračner T (2020). Screening for diabetic retinopathy—a twelve-month review. Acta Clin Croat.

[103] Chung YR, Ha KH, Lee K, Kim DJ (2020). Diabetic retinopathy and related clinical practice for people with diabetes in Korea: a 10-year trend analysis. Diabetes Metab J.

[104] Romero-Aroca P, López-Galvez M, Martinez-Brocca MA, Pareja-Ríos A, Artola S, Franch-Nadal J (2022). Changes in the epidemiology of diabetic retinopathy in Spain: a systematic review and meta-analysis. Healthcare.

[105] Agardh E, Agardh CD, Hansson-Lundblad C (1993). The five-year incidence of blindness after introducing a screening programme for early detection of treatable diabetic retinopathy. Diabet Med.

[106] Quality and Efficiency of Diabetes Care in Sweden: National Performance Assessment 2011. https://www.socialstyrelsen.se/globalassets/sharepoint-dokument/artikelkatalog/statistik/2014-3-18.pdf.

[107] van Eijk KN, Blom JW, Gussekloo J, Polak BC, Groeneveld Y (2012). Diabetic retinopathy screening in patients with diabetes mellitus in primary care: Incentives and barriers to screening attendance. Diabetes Res Clin Pract.

[108] Gillow JT, Gray JA (2001). The national screening committee review of diabetic retinopathy screening. Eye.

